# Associations between capillary glucose during pregnancy and childhood growth to the age of five: a cohort study

**DOI:** 10.1038/s41598-022-05821-8

**Published:** 2022-02-03

**Authors:** Anna Österroos, Linda Lindström, Per Wikman, Anna-Karin Wikström, Inger Sundström Poromaa, Fredrik Ahlsson

**Affiliations:** Department of Women’s and Children’s Health, Uppsala University Hospital, Uppsala University, Dag Hammarskjölds väg 14B, 1 tr, 751 85 Uppsala, Sweden

**Keywords:** Biomarkers, Risk factors, Obesity

## Abstract

The objective of this study was to evaluate the relationship between random capillary glucose levels in healthy pregnant women and infant size at birth and childhood growth to the age of five years. This population-based cohort study comprised 10,937 healthy mother–child dyads. Data on highest maternal random capillary glucose level during pregnancy and sequential anthropometric data on their children during the first five years of life were gathered from the Uppsala County Mother and Child Cohort. Statistical analyses were performed with linear regression and linear mixed effect regression models. We found that higher glucose level during pregnancy was associated with higher weight z-score (β 0.10, 95% confidence interval (CI) 0.08–0.11), length z-score (β 0.05, 95% CI 0.03–0.07) and BMI z-score (β 0.09, 95% CI 0.07–0.12) at birth, adjusted for maternal BMI and country of birth, smoking during pregnancy and parity. The association did not remain at 1½, 3, 4 and 5 years of age. There was a positive relationship between higher glucose level during pregnancy and a decrease in weight z-score, height z-score and BMI z-score from birth to 5 years of age. In conclusion, higher random capillary glucose levels in pregnant healthy women were associated with greater infant size at birth, as well as decreased growth velocity in early childhood.

## Introduction

The prevalence of childhood obesity is increasing worldwide^1^. Obesity is a great contributor to the global disease burden and causes morbidity and premature death in cardiovascular disease. The risk of obesity-associated morbidity and mortality increases for every year lived with obesity^2–4^. Children with rapid weight gain and accelerated BMI in early childhood run increased risk of sustained obesity in adult life^5^. Furthermore, children with accelerated height development during the first years of life are more prone to develop obesity in childhood^6^. Once obesity is established, it is difficult to lose weight due to homeostatic adaptations in the body^7^.

Diabetes mellitus during pregnancy is strongly associated with greater size at birth^8,9^. Good pre-gestational and gestational glycaemic control in diabetic mothers decrease the risk of macrosomia and large for gestational age (LGA) fetuses^10–12^. Further, infants to mothers with untreated mild hyperglycaemia during pregnancy have higher birth weight than infants to mothers with normal blood glucose levels during pregnancy, independent of maternal BMI, parity and maternal educational level^13^.

In addition, even infants with mothers who have not developed gestational diabetes are at higher risk of being born LGA or with macrosomia the higher maternal glucose levels during pregnancy^14–16^. So far there are conflicting results about the relationship between maternal glucose levels within normal range in pregnancy and risk of obesity in early childhood. One study investigated the growth in children from birth until 2 years of age in non-diabetic mothers. In this study, they examined the relationship between the level of randomly collected venous glucose, within normal range, in gestational week 28 and childhood growth. They observed a catch-down growth in children from birth to 24–36 weeks of post-natal age in children with mothers having a glucose level within the highest third of normal range^17^. Another study provided some evidence that maternal glucose levels within normal ranges during pregnancy correlate positively to adiposity in 10–14 year old children^18^.

Different diagnostic and screening methods are utilised for gestational diabetes (GDM). The diagnosis is based on an Oral Glucose Tolerance Test (OGTT), although cut-offs for the diagnosis differ between, and sometimes, within countries^19^. The screening procedure for gestational diabetes are either based on offering all pregnant women OGTT or a risk factor-based screening, where only those with risk factors are offered OGTT. One risk factor is a high non-fasting random capillary blood glucose level, a test that is repeatedly offered during pregnancy in all women in Swedish maternal health care^20–22^.

Until now, most studies that have investigated how glucose levels during pregnancy affect size at birth and childhood growth have been using fasting blood glucose or blood samples collected under oral glucose tolerance testing. Thus, it is not known how randomly collected capillary blood glucose levels are associated with infant size at birth or with early childhood growth until the age of 5 years. Hence, the aim of this study was to investigate the relationship between the highest level of randomly collected capillary blood glucose in healthy non-diabetic pregnant women and infant size at birth as well as growth trajectories from birth to 5 years of age.

## Results

### Participants

**Figure 1:**
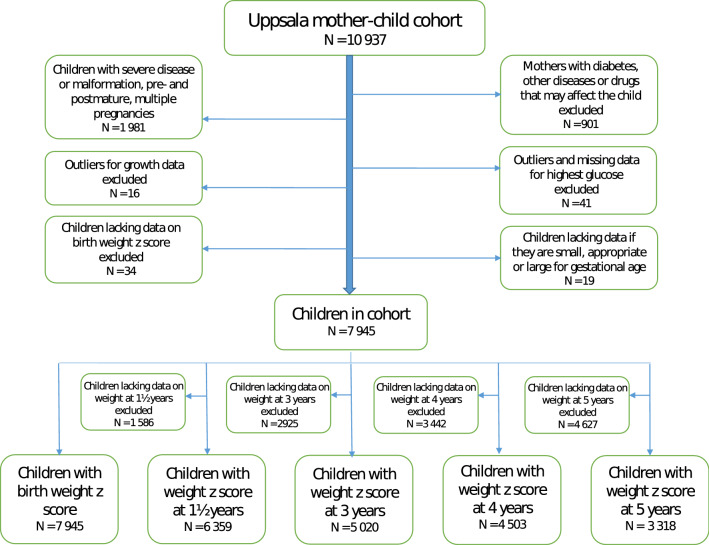
Study population. Flowchart shows causes and numbers of excluded observations and number of observations in the final study population. Number of mother–child dyads with a value of weight z-score at birth, 1½, 3, 4 and 5 years of age are found at the bottom of flow chart.

Of the 10,937 mother–child dyads in Uppsala County Mother and Child Cohort born between 2007 and 2015 we finally included 7,945 mother–child dyads (Fig. 1). Characteristics of the mother–child dyads are summarised in Table 1. Maternal mean age at child birth was 30.6 (± 5.0 SD) years and mean BMI in first trimester was 23.7 (± 4.2 SD) kg/m^2^. A majority of the mothers, 62.6% (N = 4971), had an educational level corresponding to post-secondary or university degree. Furthermore, most mothers, 84.9% (N = 6748), did not smoke at first maternal health care visit and/or in pregnancy week 30–32. Mean level of highest measured randomly collected capillary blood glucose during pregnancy was 6.2 mmol/L. Among the children, 4.4% (N = 348) were born LGA and 1.1% (N = 87) were born SGA.

**Table 1:** Characteristics of population.

Stratum of population^a^	Total population	Q1	Q2	Q3	Q4
Mother–child dyads (N)	7945	1922	1791	2161	2071
**Maternal**					
Age at pregnancy (years)	30.6 (5.0)	30.2 (4.9)	30.4 (4.9)	30.6 (4.9)	31.1 (5.1)
Primiparous (N (%))	3378 (42.5)	822 (42.8)	762 (42.5)	914 (42.3)	880 (42.5)
Weight, in first trimester (kg)^b^	66.1 (12.5)	65.3 (12.1)	65.6 (11.7)	66.1 (12.3)	67.4 (13.6)
Height (cm)	167.0 (6.4)	167.2 (6.5)	167.0 (6.3)	167.2 (6.2)	166.4 (6.3)
BMI, in first trimester (kg/m^2^)^b^	23.7 (4.2)	23.4 (4.0)	23.5 (3.9)	23.7 (4.1)	24.3 (4.5)
Education level, post-secondary education (N (%))	4971 (62.6)	1215 (63.2)	1168 (65.2)	1362 (63.1)	1226 (59.2)
**Maternal country of birth (N (%))**					
Nordic countries	7482 (94.1)	1822 (94.8)	1700 (94.9)	2047 (94.7)	1913 (92.4)
European	169 (2.1)	41 (2.1)	35 (2.0)	41 (1.9)	52 (2.5)
Other countries	294 (3.7)	59 (3.1)	56 (3.1)	73 (3.4)	106 (5.1)
**Smoking in first and/or third trimester (N (%))**^**c**^					
Smoking	390 (4.9)	106 (5.5)	83 (4.6)	92 (4.3)	109 (5.3)
Missing	807 (10.2)	205 (10.7)	191 (10.7)	200 (9.3)	211 (10.2)
Highest random capillary blood glucose mean (mmol/L)	6.2 (0.9)	5.2 (0.3)	5.8 (0.1)	6.4 (0.2)	7.5 (0.7)
25th percentile	5.6				
Median	6.1				
75th percentile	6.8				
**Offspring**					
Female sex (N (%))	3872 (48.7)	959 (49.9)	855 (47.7)	1042 (48.2)	1016 (49.1)
Birth weight (g)	3624 (476)	3554 (457)	3601 (471)	3636 (478)	3701 (485)
Large for gestational age (N (%))^d^	348 (4.4)	54 (2.8)	68 (3.8)	100 (4.6)	126 (6.1)
Small for gestational age (N (%))^d^	87 (1.1)	23 (1.2)	26 (1.5)	25 (1.2)	13 (0.6)

*N*  numbers of observations. Data represent the mean (SD), unless otherwise indicated.

^a^Total population and population divided by quartiles of highest maternal random capillary blood glucose level during pregnancy. Q1 = glucose quartile 1, Q2 = glucose quartile 2, Q3 = glucose quartile 3, Q4 = glucose quartile 4.

^b^First visit to maternal health care service in pregnancy week 9–12.

^c^Smoking at first maternal health care service in pregnancy week 9–12 and/or in pregnancy week 30–32.

^d^Large for gestational age defined as weight + 2 SD and small for gestational age defined as weight −2 SD at birth. Marsal’s intrauterine growth curves as reference standards for growth data for new-borns.

Mean birth weight was 3624 (± 476 SD) g and mean birth length was 50.9 (± 2.0 SD) cm. A decrease in weight, height and BMI z-score was seen from birth to five years of age. Mean weight z-score was 0.12 (± 0.9 SD) at birth and −0.08 at 5 years of age. Length z-score at birth was 0.08 (± 0.9 SD) whilst we observed the lowest level −0.02 (± 1.0 SD) of height z-score at 3 years of age. BMI z-score was 0.13 (± 0.9 SD) at birth and −0.11 (± 0.9 SD) at 5 years of age. Of all 5-year-old children, 10.9% (N = 362) were overweight and 2.2% (N = 74) were obese (Table 2).

**Table 2:** Characteristics of children.

Overall (N)	7945				
Age at follow up (years)	Birth	1½	3	4	5
Weight (kg)	3.6 (0.5)	11.7 (1.3)	15.3 (1.8)	17.5 (2.1)	19.7 (2.7)
Weight-z score^a^	0.1 (0.9)	0.0 (1.0)	−0.1 (1.0)	−0.1 (1.0)	−0.1 (0.9)
Height (cm)	50.9 (2.0)	83.0 (2.9)	96.3 (3.7)	104.3 (4.1)	111.5 (4.5)
Height-z score^a^	0.1 (0.9)	−0.0 (1.0)	−0.0 (1.0)	−0.0 (1.0)	−0.0 (1.0)
BMI (kg/m^2^)	13.9 (1.3)	16.9 (1.4)	16.4 (1.3)	16.0 (1.3)	15.8 (1.4)
BMI-z-score^a^	0.1 (0.9)	0.0 (1.0)	−0.0 (1.0)	−0.1 (1.0)	−0.1 (0.9)
**Overweight and obesity**					
Overweight, total (N (%))^b^	–	–	648 (12.9)	479 (10.6)	362 (10.9)
Obese, total (N (%))^b^	–	–	96 (1.9)	86 (1.9)	74 (2.2)
Overweight girls (N (% of girls))^b^	–	–	325 (13.3)	235 (10.6)	188 (11.7)
Obese girls (N (% of girls))^b^	–	–	48 (2.0)	50 (2.3)	47 (2.9)
Overweight boys (N (% of boys))^b^	–	–	323 (12.5)	244 (10.7)	174 (10.2)
Obese boys (N (% of boys))^b^	–	–	48 (1.9)	36 (1.6)	27 (1.6)

*N*  numbers of observations. Data represent the mean (SD), unless otherwise indicated.

^a^Sex and age independent standard deviation scores (z-scores) were calculated using the Swedish reference population.

^b^Revised international (International Obesity Task Force; IOTF) BMI cut‐offs (kg/m^2^) using the pooled LMS curves.

Data on the first measured glucose level were available in 48.8% of the mother–child dyads (Supplementary Table S4). Out of these a majority (80.2%) of the highest measured random capillary blood glucose levels were collected in pregnancy week 25 or later whereas a fifth (19.8%) were collected before pregnancy week 13.

### Glucose and outcome

#### Linear analyses

The highest capillary blood glucose level during pregnancy was significantly associated with an increase in birth weight z-score, length z-score and BMI z-scores (p < 0.001), (Table 3). For every increase in maternal blood glucose level of 1 mmol/L, the birth weight z-score increased 0.10 (95% CI 0.08–0.12), birth length z-score increased 0.05 (95% CI 0.03–0.07) and BMI z-score at birth increased 0.09 (95% CI 0.07–0.12) in the adjusted models. At 1½ years we observed an inverse relationship between maternal glucose level during pregnancy and weight z-score (p < 0.036). For every 1 mmol/L increase in maternal blood glucose level the weight z-score at 1½ years decreased with 0.03 (95% CI −0.05 to −0.00) in the adjusted model. At all other ages there was no relationship between the maternal blood glucose level during pregnancy and anthropometric data of the child in the adjusted models. We ran similar analyses on imputed datasets for smoking in pregnancy week 30–32 derived from the original cohort since 8.4% of individuals were missing data on this variable. There were no differences in results when compared the individuals with incomplete data on smoking.

**Table 3:** Associations between growth data expressed as weight z score, height z score and BMI z score from birth to five years of age and highest random capillary blood glucose level during pregnancy.

	Univariate model			Adjusted model^a^		
	*N*	β (95% CI)	p-value	*N*	β (95% CI)	p-value
**Weight-z score**						
Birth	7945	0.11 (0.09–0.13)	< 0.001*	7913	0.10 (0.08–0.11)	< 0.001*
1½ year	6359	−0.02 (−0.05 to 0.01)	0.12	6335	−0.03 (−0.05 to −0.00)	0.04*
3 years	5020	0.00 (−0.03 to 0.03)	0.93	4997	−0.01 (−0.04 to 0.02)	0.60
4 years	4503	0.00 (−0.03 to 0.03)	0.77	4481	−0.01 (−0.04 to 0.02)	0.59
5 years	3318	0.00 (−0.03 to 0.04)	0.80	3304	−0.01 (−0.04 to 0.02)	0.61
**Height-z score**						
Birth	7911	0.06 (0.04–0.08)	< 0.001*	7879	0.05 (0.03–0.07)	< 0.001*
1½ year	6361	−0.01 (−0.03 to 0.02)	0.58	6337	−0.01 (−0.04 to 0.01)	0.38
3 years	5022	0.00 (−0.03 to 0.03)	0.97	4999	−0.00 (−0.03 to 0.03)	0.94
4 years	4505	0.00 (−0.03 to 0.03)	0.79	4483	−0.00 (−0.03 to 0.03)	0.94
5 years	3319	−0.01 (−0.05 to 0.02)	0.47	3305	−0.01 (−0.05 to 0.02)	0.48
**BMI-z score**						
Birth	7910	0.11 (0.08–0.13)	< 0.001*	7878	0.09 (0.07–0.12)	< 0.001*
1½ year	6351	−0.02 (−0.04 to 0.01)	0.20	6327	−0.02 (−0.05 to −0.00)	0.08
3 years	5019	0.00 (−0.03 to 0.03)	0.82	4996	− 0.01 (−0.04 to 0.02)	0.61
4 years	4500	0.00 (−0.03 to 0.03)	0.81	4478	− 0.01 (−0.04 to 0.02)	0.44
5 years	3318	0.02 (−0.01 to 0.05)	0.28	3304	−0.00 (−0.03 to 0.03)	0.96

Analyses performed with univariate and multivariate linear regression.

Sex and age independent standard deviation scores (z-scores) were calculated using the Swedish reference population.

*N*  number of observations, *β*  β-value, *95% CI* 95% confidence Interval.

*p < 0.05.

^a^Adjusted for mothers' BMI at first maternal care visit, maternal country of birth, parity, smoking at first visit at maternal health care and/or in pregnancy week 30–32.

#### Linear mixed effect model analyses

**Figure 2:**
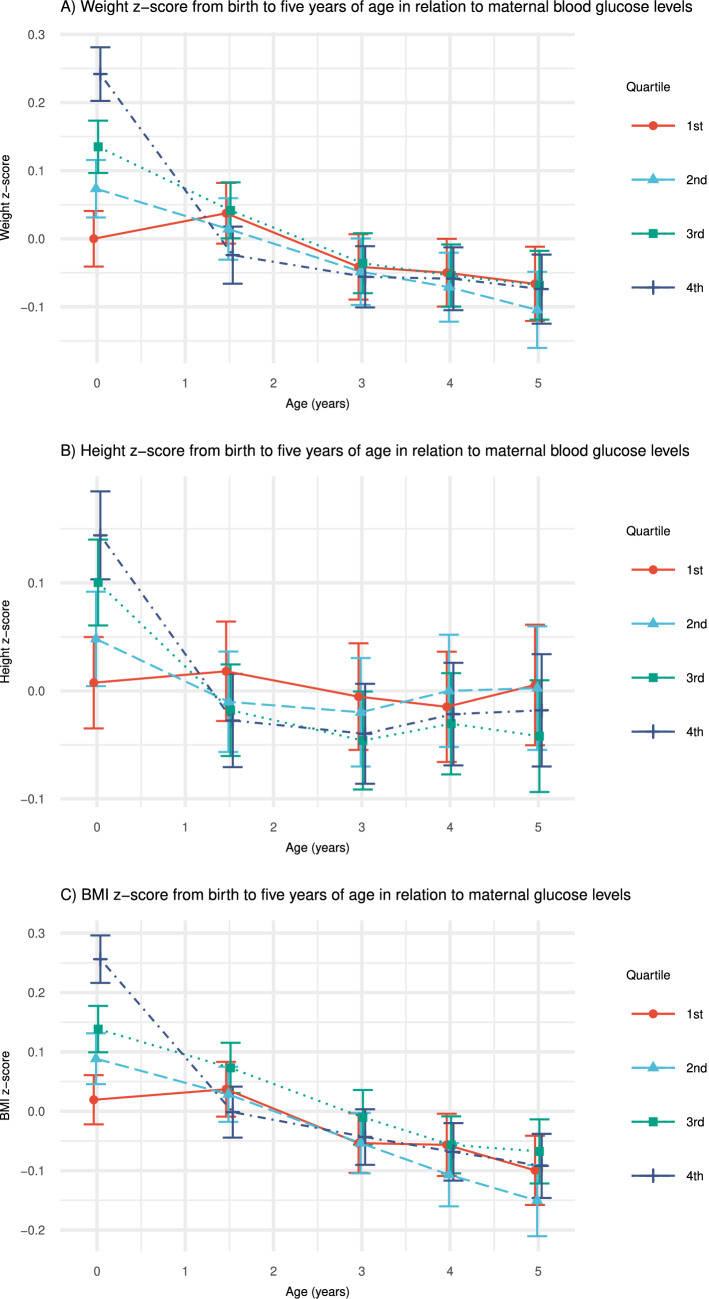
Child's growth from birth to 5 years of age in relation to highest maternal random capillary blood glucose level during pregnancy. Analyses performed with linear mixed effect model with glucose divided into quartiles. **(A)** Child's weight-z score, **(B)** height z-score and **(C)** BMI z-score from birth to 5 years of age in relation to highest maternal random capillary blood glucose level during pregnancy divided into quartiles. Bars represent 95% confidence intervals.

The range of weight z-score, height z-score and BMI z-score, between lowest and highest glucose quartile, were larger at birth than at 1½, 3, 4 and 5 years of age (Fig. 2 and Supplementary Tables S1–S3). We showed a positive relationship between maternal glucose quartiles and weight z-score, height z-score and BMI z-score at birth. Compared with children born to mothers with glucose level within quartile one (Q1), children to mothers with glucose level within quartile two (Q2), three (Q3) and four (Q4) had significantly higher weight and BMI z-scores at birth. Further, those with glucose level within Q3 and Q4 had higher height z-score at birth (Supplementary Tables S1–3).

We found an inverse relationship between maternal glucose quartiles and weight z-score growth trajectory from birth to 1½, 3, 4 and 5 years of age. The decrease in weight z-score were least in children to mothers with glucose level within Q2 and greatest for Q4 compared with children to mothers with glucose level within Q1 (Supplementary Table S1).

We found inverse relationships between glucose quartiles and growth trajectories, with a greater decrease in height z-score for children to mothers with glucose level within Q2 from birth to 1½ years and for Q3 and Q4 from birth to 3, 4 and 5 years compared with children to mothers with glucose level within Q1 (Supplementary Table S2). We found no difference in height growth trajectories between children to mothers with glucose level within Q2 compared to children to mothers with glucose level within Q1.

Greater decreases in BMI z-scores were noted in children to mothers with glucose level within Q2, Q3 and Q4 from birth to 1½ years of age compared with children to mothers with glucose level within Q1. Furthermore, there was a greater decrease in BMI z-score for children to mothers with glucose level within Q3 and Q4 from birth to 3 years and for Q2, Q3 and Q4 from birth to 4 and 5 years of age compared with children to mothers with glucose level within Q1 (Supplementary Table S3). We found no difference BMI z-scores between children to mothers with glucose level within Q2 compared to children to mothers with glucose level within Q1 from birth to 3 years of age.

After adjustment for the potential confounders parity, maternal BMI in first trimester, maternal country of birth and smoking at first maternal health care visit and/or in pregnancy week 30–32 the results did not change. We performed similar analyses on imputed datasets for smoking at first maternal health care visit and/or in pregnancy week 30–32 and there were no differences in results compared with individuals with incomplete data on smoking.

## Discussion

In this study, we show that a higher level of randomly collected capillary blood glucose in healthy pregnant women is associated with higher infant weight, height and BMI z-scores at birth. In addition, we show that children of mothers within the highest glucose quartile had the greatest decrease in weight, height and BMI z-scores from birth to 1½, 3, 4 and 5 years of age. However, there was no relationship between the highest glucose level during pregnancy and size at 1½, 3, 4 or 5 years of age. The new finding in this study is that we could demonstrate a positive association between the highest measured random capillary glucose level within normal glucose range, after multiple measurements during pregnancy, and a decrease in weight, height and BMI z-score from birth until 5 years of age in a large population based cohort of mother-and-child dyads.

Our results are consistent with earlier studies, where fasting, not random glucose levels during pregnancy in mothers with and without diabetes correlate positively to fetal growth and birth size^10–12,15,16^. Also mild hyperglycaemia during pregnancy in non-diabetic mothers lead to increased birth weight and the association between maternal glucose level and birth weight seems to be linear^23^. Regarding growth trajectories in early childhood, earlier studies demonstrates a catch-down growth from birth to a few months of age in children to diabetic mothers^24,25^. Even in children to non-diabetic mothers the growth pattern of catch-down growth in children to mothers with the highest glucose levels, within normal range, has been observed^17^. As in our study, Stenhouse et al. showed a catch-down growth in children to non-diabetic mothers from birth to 6–9 months of age that correlated with the highest thirtile, within normal range, of randomly collected venous glucose levels in pregnancy week 28. We have thus replicated their finding, and also extended it, by showing this relationship is independent of maternal BMI and parity^17^.

Among existing studies on the association between glucose levels during pregnancy in non-diabetic mothers and risk of childhood obesity there are conflicting results. In two different studies Hillier et al. and Deierlein et al. showed an association between glucose levels during pregnancy in non-diabetic mothers and risk of early childhood obesity^26,27^. One of these studies adjusted for maternal BMI and the other one did not. On the contrary, an observational multicentre study by Thaware et al. found such an association prior adjusting for maternal pre-pregnancy BMI but not after adjustment^28^. In a study by Gomes et al. children to obese mothers with dysglycemia in third trimester, related to excessive gestational weight gain, had a higher BMI at birth, a greater weight gain in early childhood and higher BMI at 4 years of age than children to obese mothers without dysglycemia^42^. Although there are conflicting results about the influence of maternal glucose levels during pregnancy in non-diabetic mothers and risk of childhood obesity the gathered evidence suggest that maternal BMI is an important factor for childhood size and growth and therefore important to adjust for when examining childhood growth in relation to maternal glucose levels during pregnancy. In this study, we adjusted for maternal BMI in the first trimester and the results remained unaltered after the adjustment.

The proportion of overweight and obese children is steadily increasing worldwide causing both morbidity and preterm death^4,5^. In our study population, the proportion of overweight and obesity were similar to the prevalence of overweight and obesity among 4 year old children in Sweden^29^. This makes our data reliable and generalizable despite our study population consisted of healthy mothers and children. In a large study, with pooled data from about 2400 population based studies including 5–19 year old children, the prevalence of overweight and obesity in North-western Europe were a few percentage points higher compared to the prevalence of overweight and obesity in our study population. However, since the prevalence of overweight and obesity is increasing with age, the prevalence of overweight and obesity is expected to be lower in our population than in studies including older children^30^. Every piece that contributes to the understanding of childhood growth and development of obesity is beneficial for future efforts to prevent children from developing obesity. Our findings suggest that maternal glucose level during pregnancy is one prenatal factor contributing to the childhood growth pattern where higher glucose level is associated with a decreased growth velocity during first years of life but not actual size at the age of five.

A catch-down growth pattern is often seen in children born LGA^31^. Being born LGA is associated with higher cardio-metabolic risk later in life. Children born LGA with a small catch-down growth seem to have lower risk of cardio-metabolic outcomes compared with children born LGA without a catch-down growth whom seem to have an increased risk of cardio-metabolic outcomes^32,33^. Our data suggests that children to mothers with glucose levels within the higher normal interval are larger at birth and, as many children born LGA, tend to have a lower growth velocity during the first years compared with children to mothers with glucose levels in the lower normal range.

Earlier studies have focused on glucose values from oral glucose tolerance tests, fasting samples or randomly collected venous samples. In Swedish maternal health care, the routine is to take random capillary blood glucose samples that makes our approach more applicable to the Swedish healthcare system than other forms of glucose tests and may be easy to apply in other maternal health care systems as well. Furthermore, earlier studies have focused on a specific time in pregnancy for the glucose analysis whereas our study have selected the highest measured glucose level out of multiple measurements throughout pregnancy. This knowledge could guide maternal health care professionals to inform pregnant women about the importance of physical activity and a healthy diet whenever a glucose level in the higher normal range is measured during pregnancy^34,35^.

A strength of this study was the large cohort of 10,937 children. This allowed us to exclude children with potential risk of abnormal postnatal growth. Moreover, we had a relatively long follow up time compared with other studies of maternal glucose levels during pregnancy and growth patterns of children during early childhood. Still it would have been valuable to follow the children even longer to study whether there is an association between maternal glucose levels during pregnancy and the children’s growth trajectories and anthropometry when they turn into adolescents. This may be an issue for future investigations.

Another strength of this cohort study was that we collected data from Swedish Medical Birth Register, which is regarded as a high quality registry, which increases the credibility of the results of this study^36^. Also the high attendance of the Swedish population in child health care services makes data from the Database of statistics of the Child Health Care Unit in Uppsala County credible. The growth charts that we used are made from two Swedish study populations, which ought to make them consistent with our study population since almost 94% of mothers were born in Nordic countries. The low prevalence of gestational diabetes in this study is in line with previous studies from Sweden^37^.

A limitation of this study was that we did not have information on in which gestational week the highest glucose level is noted in each individual. This makes it hard to draw any conclusions about the importance of the timing of the glucose sample when estimating the relationship between maternal glucose level and size and growth of the child. The insulin sensitivity changes during pregnancy and decrease when pregnancy is proceeding. Secretion of insulin increases from second trimester until delivery along with the decreasing insulin sensitivity^38,39^. These changes are accompanied with a decrease in fasting glucose level later in pregnancy and a higher post prandial glucose level that is maintained for a longer period^40,41^. Depending on when in pregnancy a random capillary glucose sample is taken the level may vary, with lower values to be expected in early pregnancy and higher levels in late pregnancy. As expected, a majority (80.2%) of the highest random capillary blood glucose values measured in our population were collected in pregnancy week 25 or later. Nevertheless, a fifth (19.8%) of the random capillary blood glucose levels were collected before pregnancy week 13. Our data suggests that it may not matter when in pregnancy you measure a high glucose value within normal range; a higher glucose level measured at any time in pregnancy, in a non-diabetic mother, is associated with a greater size at birth and a decreased growth velocity in early childhood. Since blood glucose samples were random collected and not fasting samples there could be non-differential misclassification. Another limitation of the study is the limited information on potential confounders. We have used educational level as a proxy for socioeconomic status. Unfortunately we do not have any information on maternal or child diet and behavioural factors, such as physical activity, which might have an impact on fetal and childhood growth. Further we do not have information on anthropometry at age 2 years.

We saw a growth pattern with a greater size at birth and a decrease in weight and BMI z-score until 5 years of age. Based on our data we cannot know the reason for this. Though we think that the greater size at birth and the decreased size at 5 years can be a result of our selected population of healthy children born at term to healthy mothers. Diseases that may affect the growth of the offspring were excluded, e.g. preeclampsia and children with malformations and chromosome aberrations. Another contributing factor may be our relatively highly educated population, Uppsala County Mother and Child Cohort, because Uppsala is a University City and the educational level is higher compared with the national average. In general, high educational level is associated with a healthier lifestyle and the growth from birth until the age of five is highly influenced by the lifestyle in the family.

## Conclusions

Randomly collected capillary glucose levels during pregnancy in non-diabetic mothers correlate with infant size at birth and a decrease in weight, height and BMI z-scores in early childhood. However, there were no associations between the maternal glucose levels during pregnancy and anthropometry at 1½, 3, 4 and 5 years of age. Factors others than glucose levels during pregnancy may be of greater importance for size in early childhood.

## Methods

### Data sources

This study is based on data from The Uppsala County Mother and Child (UCMC) Cohort, a population-based cohort originally based on a local register for the Child Health Care in Uppsala County. The database comprise nearly 60,000 children born at Uppsala University Hospital, between 2000 and 2015. The UCMC Cohort use data from the *Database of statistics of the Child Health Care Unit in Uppsala County*, which holds information on repeated anthropometric measurements (birth, 1½ years, 3 years, 4 years and 5 years) and health data (referrals, vaccinations). Children living in Uppsala County have an attendance in child health care of 97%^43,44^.

Further, the UCMC Cohort database incorporates data from a number of the Swedish health registries, including the Swedish *Medical Birth Register (SMBR) and* the *Registry of Education* (parental level of education)^45^, the *Registry of Total Population* (maternal country of birth), the *Causes of Death Registry* (annual data on time and causes of death). Finally, the UCMC Cohort holds data from the *Inpatient Care Regist*ry, containing parental and child discharge diagnoses, covering all private and public hospitalizations, and the *Prescribed Drug Registry* with information on all prescribed drugs dispatched in Swedish pharmacies. All Swedish residents allocate a unique national registration number. With use of these national registration numbers, data from the different registers were linked.

The Swedish *Medical Birth Register* is a nationwide register that comprises prospectively collected data of about 99% of all pregnancies in Sweden. The maternal, delivery and child health care services in Sweden provide standardized schedules and are free of charge. Every health care provider is compelled to report information about all pregnancies, with a duration of 22 + 0 weeks or longer, to SMBR^36^. Complications and diseases are classified and coded according to the International Classification of Diseases (ICD). SMBR applies Marsal’s intrauterine growth curves as reference standards for growth data for new-borns^46^. Children with weight or length + /− 2 standard deviations from mean of reference population for new-borns are defined as large for gestational age (LGA) and small for gestational age (SGA), respectively^47,48^.

The first antenatal visit in maternal health care usually takes place before gestational week 13 and includes an interview with questions about the medical and obstetric history as well as social situation including smoking habits. Weight and height are measured and randomly collected capillary blood glucose is analysed. Gestational age is predominantly estimated with ultrasonography in first or second trimester of pregnancy^49^. In pregnancy week 30–32, mothers are asked a second time about smoking habits. Randomly collected capillary blood glucose is analysed at maternal health care visits in pregnancy weeks 25, 28–29, 33 and 37.

### Study population

This study includes information from the UCMC Cohort of 10,937 mother–child dyads with infants born between 2007 and 2015. Children born preterm (gestational age less than 37 + 0, or 259 days) and postterm (gestational age more than 41 + 6, or 293 days) were excluded (n = 1,012). We excluded children born after multiple pregnancies (n = 855), and children lacking data on birth weight z-score and SGA or LGA status (n = 34 and n = 19, respectively). To decrease the risk of impaired growth caused by diseases in the children, we excluded children with chromosome aberrations and congenital malformations (n = 59). Individuals with data on size (weight z-score, height z-score and BMI z-score) at birth, 1½, 3, 4 and 5 years of age less than −5 or more than + 5 standard deviations (SD) from mean were regarded as misclassified and excluded (n = 16). Furthermore, children of mothers with inflammatory or autoimmune disease, essential and gestational hypertension, preeclampsia, cardiovascular disease, hyperthyroidism and epilepsy were excluded (n = 901). Children with mothers diagnosed with pregestational diabetes and gestational diabetes were excluded (n = 125). Mothers with missing data for highest glucose level (n = 39) and mothers with a highest value of randomly collected capillary blood glucose > 12.1 mmol/L and their children were excluded due to potential risk of maternal diabetes mellitus (n = 2). The final population consisted of 7945 mother–child dyads.

### Exposure

The highest measured randomly collected non-fasting capillary blood glucose level in mmol/l obtained at any time throughout the pregnancy was the exposure in the study. The value of the first glucose sample, collected before pregnancy week 13, were included in the data collection for statistical analyses. Glucose samples were collected capillary by midwives, as a part of routine maternal health care and analysed on point-of-care testing instruments at the maternal health unit. The instruments are provided and validated by Uppsala University Hospital laboratory and are routinely checked and calibrated to secure quality of analyses. The pregnant women were not told to be fasting before sampling. The blood samples were collected at any given point in the day and at any time in relation to meals which is the clinical practise in Sweden. In Uppsala screening with OGTT for gestational diabetes is based on risk factors, not offered to all women. Mothers with a randomly collected capillary blood glucose level ≥ 8.8 mmol/L, mothers with a BMI ≥ 35 kg/m^2^, mothers with a previous history of gestational diabetes or having a child born LGA or mothers with parents or siblings with diabetes mellitus type 2 underwent OGTT. Furthermore, OGTT was also carried out in mothers with suspected increased fetal growth based on symphyseal fundal height, weight estimated to >  + 22% or polyhydramnios at ultrasonography. Pregnant women that underwent OGTT with a fasting plasma glucose level ≥ 7.0 mmol/L or a plasma glucose level ≥ 9.0 mmol/L 120 min after glucose intake were diagnosed with gestational diabetes (GDM), and excluded from this study. Data about maternal glucose levels were gathered from maternal records and linked with register data.

### Outcome

Offspring growth expressed as weight z-score, height z-score and BMI z-score at birth, 1½, 3, 4 and 5 years were the outcomes. Swedish reference curves are used for documentation of anthropometric data in child health care, and standard deviation scores (z-scores) were calculated using the Swedish reference population^48,50^. The child’s BMI was calculated with weight in kg with two decimals at birth and at 1½ years and weight in kg with one decimal at 3, 4 and 5 years of age and height in metres with two decimals for all ages. Only children with anthropometric values collected two months before or after 1½, 3, 4 and 5 years of age were included.

### Covariates

To select covariates for making adjustments for potential confounders in analyses we drew a directed acyclic graph (DAG) (supplementary Fig. S1). We adjusted for the covariates parity, maternal BMI at first antenatal visit at maternal health care, maternal country of birth and smoking at first maternal health care visit and/or in pregnancy week 30–32 as suggested by the DAG for minimal sufficient adjustment set. When calculating maternal BMI we used weight in whole kg and height in metres with two decimals. Maternal country of birth was divided into three categories; born in a Nordic country, European country or a non-European country. Smoking was divided into no smoking and smoking.

### Statistical analyses

For baseline characteristics, we presented quantitative variables as the mean (± SD) of study population, and categorical variables as proportions (percentages). We made separate univariate and multivariate linear regression analyses for highest random capillary glucose level during pregnancy and the outcomes child weight z-score, height z-score and BMI z-score for ages 0 (birth), 1½, 3, 4 and 5 years, respectively. Results were expressed as beta coefficients with 95% CI.

In order to take repeated measurements into account and to examine the growth trajectories of the children, we performed linear mixed effect regression model analyses. We divided the children into quartiles according to the highest level of their mothers’ random capillary blood glucose during pregnancy. The first glucose quartile (Q1) included children to mothers with random capillary blood glucose level < 5.6 mmol/L, the second quartile (Q2) 5.6–6.0 mmol/L, the third quartile (Q3) 6.1–6.7 mmol/L and the fourth quartile (Q4) ≥ 6.8 mmol/L. We performed separate linear mixed regression model analyses where we compared the weight z-score, height z-score and BMI z-score trajectories of the glucose quartile groups from birth to the follow-up ages 1½, 3, 4 and 5 years. In the analyses, we handled each child (e.g. subject) as random effect to allow the intercept to vary by child. Age and glucose quartiles were handled as fixed effects as well as the covariates parity, maternal BMI, maternal country of birth and smoking. For all interactions between maternal glucose quartiles and age of the children, we used glucose Q1 as reference value. Growth trajectories from birth to the four follow-up points (e.g. 1½, 3, 4 and 5 years of age) for children to mothers with glucose levels within Q2, Q3 and Q4 were compared with the growth trajectory of children to mothers with glucose level within Q1. We presented the results as beta coefficients with 95% CI for fixed effects.

Due to a large proportion of missing values for smoking at first maternal health care visit and/or in pregnancy week 30–32 (11.3%) we conducted supplementary analyses after multiple imputation by chained equations. We imputed data for smoking at first maternal health care visit and/or in pregnancy week 30–32 by using the child’s birth weight, highest maternal level of education and highest measured glucose level during pregnancy.

Statistical significance for all analyses were set to *p* < 0.05. We conducted all statistical analyses with R version 3.5.3 (R Core Team, 2019)^51^ packages lme4^52^, tidyr^53^, mice^54^ and figures were produced using package ggplot2^55^.

### Ethics

The Regional Ethical Review Board in Uppsala attested ethic permission of the project (no. 2014/353). This study was performed in accordance with national and international guidelines for medical research. Statistics Sweden merged and de-identified the registry data of Uppsala County Mother and Child Database before delivery to the research group. Since participants were neither identified nor contacted, informed consent was not required. The need of informed consent was waived by the Regional Ethical Review Board in Uppsala. When a study has been approved by a relevant ethics committee, informed consent is not requisite in Sweden and other Nordic countries for large registry-based studies in general^56^.

## Supplementary Information

Supplementary Information.

## Data Availability

The datasets generated during and/or analysed during the current study are available from the corresponding author on reasonable request.

## References

[1] (NCD-RisC), N.R.F.C. Worldwide trends in body-mass index, underweight, overweight, and obesity from 1975 to 2016: A pooled analysis of 2416 population-based measurement studies in 128.9 million children, adolescents, and adults. *Lancet (London, England)* **390**, 2627–2642 (2017). 10.1016/S0140-6736(17)32129-3 PMC5735219 29029897

[2] Abdullah A (2011). The number of years lived with obesity and the risk of all-cause and cause-specific mortality. Int. J. Epidemiol..

[3] Twig, G.* et al.* Body-mass index in 2.3 million adolescents and cardiovascular death in adulthood. *N. Engl. J. Med.* **374**, 2430–2440 (2016). 10.1056/NEJMoa1503840 27074389

[4] Park MH, Falconer C, Viner RM, Kinra S (2012). The impact of childhood obesity on morbidity and mortality in adulthood: A systematic review. Obesity Rev..

[5] Geserick M (2018). Acceleration of BMI in early childhood and risk of sustained obesity. N. Engl. J. Med..

[6] Papadimitriou, A., Nicolaidou, P., Fretzayas, A. & Chrousos, G.P. Clinical review: Constitutional advancement of growth, a.k.a. early growth acceleration, predicts early puberty and childhood obesity. *J. Clin. Endocrinol. Metab.* **95**, 4535–4541 (2010). 10.1210/jc.2010-0895 20610589

[7] MacLean PS, Higgins JA, Giles ED, Sherk VD, Jackman MR (2015). The role for adipose tissue in weight regain after weight loss. Obesity Rev..

[8] Landon MB (2015). Mild gestational diabetes mellitus and long-term child health. Diabetes Care.

[9] Rodrigues S, Ferris AM, Peréz-Escamilla R, Backstrand JR (1998). Obesity among offspring of women with type 1 diabetes. Clin. Invest. Med. Med. Clin. Exp..

[10] Gold AE, Reilly R, Little J, Walker JD (1998). The effect of glycemic control in the pre-conception period and early pregnancy on birth weight in women with IDDM. Diabetes Care.

[11] Raychaudhuri K, Maresh MJ (2000). Glycemic control throughout pregnancy and fetal growth in insulin-dependent diabetes. Obstet. Gynecol..

[12] Langer O (1989). Glycemic control in gestational diabetes mellitus–how tight is tight enough: Small for gestational age versus large for gestational age?. Am. J. Obstet. Gynecol..

[13] Vambergue A (2000). Is mild gestational hyperglycaemia associated with maternal and neonatal complications? The Diagest Study. Diabetic Med..

[14] Tallarigo L (1986). Relation of glucose tolerance to complications of pregnancy in nondiabetic women. N. Engl. J. Med..

[15] Farmer G (1988). The influence of maternal glucose metabolism on fetal growth, development and morbidity in 917 singleton pregnancies in nondiabetic women. Diabetologia.

[16] Sermer, M.*, et al.* Impact of increasing carbohydrate intolerance on maternal-fetal outcomes in 3637 women without gestational diabetes. The Toronto Tri-Hospital Gestational Diabetes Project. *Am. J. Obstet. Gynecol.* **173**, 146–156 (1995). 10.1016/0002-9378(95)90183-3 7631672

[17] Stenhouse E, Wright DE, Hattersley AT, Millward BA (2006). Maternal glucose levels influence birthweight and 'catch-up' and 'catch-down' growth in a large contemporary cohort. Diabetic Med..

[18] Lowe WL (2019). Maternal glucose levels during pregnancy and childhood adiposity in the hyperglycemia and adverse pregnancy outcome follow-up study. Diabetologia.

[19] (WHO), W.H.O. *World Health Organization Diagnostic Criteria and Classification of Hyperglycaemia First Detected in Pregnancy*. (2013). 24199271

[20] Ostlund I, Hanson U (2004). Repeated random blood glucose measurements as universal screening test for gestational diabetes mellitus. Acta Obstet. Gynecol. Scand..

[21] Berg M, Adlerberth A, Sultan B, Wennergren M, Wallin G (2007). Early random capillary glucose level screening and multidisciplinary antenatal teamwork to improve outcome in gestational diabetes mellitus. Acta Obstet. Gynecol. Scand..

[22] Hautala L, Englund E, Turkmen S (2019). Performance of variables in screening for gestational diabetes. Eur. Endocrinol..

[23] Metzger BE (2008). Hyperglycemia and adverse pregnancy outcomes. N. Engl. J. Med..

[24] Touger L (2005). Early growth in offspring of diabetic mothers. Diabetes Care.

[25] Dode MA, Santos IS, González DA (2011). Anthropometry from birth to 24 months among offspring of women with gestational diabetes: 2004 Pelotas birth cohort. J. Dev. Orig. Health Dis..

[26] Hillier TA (2007). Childhood obesity and metabolic imprinting: The ongoing effects of maternal hyperglycemia. Diabetes Care.

[27] Deierlein AL, Siega-Riz AM, Chantala K, Herring AH (2011). The association between maternal glucose concentration and child BMI at age 3 years. Diabetes Care.

[28] Thaware PK (2015). Untreated mild hyperglycemia during pregnancy and anthropometric measures of obesity in offspring at age 5–7 years. Diabetes Care.

[29] J. Miregard, C.N. *Övervikt och fetma bland 4-åringar i Sverige*. https://samverkan.regionsormland.se/for-vardgivare/halsoval/barnhalsovard/rapporter-fran-barnhalsovarden-sormland/ (2020). 34637127

[30] Wijnhoven TM (2014). WHO European Childhood Obesity Surveillance Initiative: body mass index and level of overweight among 6-9-year-old children from school year 2007/2008 to school year 2009/2010. BMC Public Health.

[31] Taal HR, Vd Heijden AJ, Steegers EA, Hofman A, Jaddoe VW (2013). Small and large size for gestational age at birth, infant growth, and childhood overweight. Obesity (Silver Spring, Md).

[32] Lei X (2018). Childhood health outcomes in term, large-for-gestational-age babies with different postnatal growth patterns. Am. J. Epidemiol..

[33] Renom Espineira A (2011). Postnatal growth and cardiometabolic profile in young adults born large for gestational age. Clin. Endocrinol..

[34] Antoun, E.*, et al.* Maternal dysglycaemia, changes in the infant's epigenome modified with a diet and physical activity intervention in pregnancy: Secondary analysis of a randomised control trial. *PLoS Med.* **17**, e1003229 (2020). 10.1371/journal.pmed.1003229 PMC7643947 33151971

[35] Rasmussen, L.*, et al.* Diet and healthy lifestyle in the management of gestational diabetes mellitus. *Nutrients* **12**, 68 (2020). 10.3390/nu12103050 PMC7599681 33036170

[36] Källén, B.K., Karin. *The Swedish Medical Birth Register—A Summary of Content and Quality*. http://www.socialstyrelsen.se/NR/rdonlyres/E9BE4DDE-95EE-4E3F-A56F-36CA5125CA8C/1132/20031123.pdf (2003).

[37] Ahlsson F, Lundgren M, Tuvemo T, Gustafsson J, Haglund B (2010). Gestational diabetes and offspring body disproportion. Acta Paediatr. (Oslo, Norway: 1992).

[38] Catalano PM, Tyzbir ED, Roman NM, Amini SB, Sims EA (1991). Longitudinal changes in insulin release and insulin resistance in nonobese pregnant women. Am. J. Obstet. Gynecol..

[39] Catalano PM (1993). Carbohydrate metabolism during pregnancy in control subjects and women with gestational diabetes. Am. J. Physiol..

[40] Catalano PM (1992). Longitudinal changes in basal hepatic glucose production and suppression during insulin infusion in normal pregnant women. Am. J. Obstet. Gynecol..

[41] Di Cianni G, Miccoli R, Volpe L, Lencioni C, Del Prato S (2003). Intermediate metabolism in normal pregnancy and in gestational diabetes. Diabetes Metab. Res. Rev..

[42] Gomes, D.*, et al.* Late-pregnancy dysglycemia in obese pregnancies after negative testing for gestational diabetes and risk of future childhood overweight: An interim analysis from a longitudinal mother-child cohort study. *PLoS Med.* **15**, e1002681 (2018). 10.1371/journal.pmed.1002681 PMC6205663 30372451

[43] Wettergren, B., Blennow, M., Hjern, A., Söder, O. & Ludvigsson, J.F. Child health systems in Sweden. *J. Pediatr.* **177s**, S187–S202 (2016). 10.1016/j.jpeds.2016.04.055 27666267

[44] Wallby, T. & Hjern, A. Child health care uptake among low-income and immigrant families in a Swedish county. *Acta Paediatr. (Oslo, Norway: 1992)* **100**, 1495–1503 (2011). 10.1111/j.1651-2227.2011.02344.x 21535134

[45] Statistics Sweden. *Educational Attainment of the Population*. https://www.scb.se/en/finding-statistics/statistics-by-subject-area/education-and-research/education-of-the-population/educational-attainment-of-the-population/ (2020).

[46] Marsál, K.*, et al.* Intrauterine growth curves based on ultrasonically estimated foetal weights. *Acta Paediatr. (Oslo, Norway: 1992)* **85**, 843–848 (1996). 10.1111/j.1651-2227.1996.tb14164.x 8819552

[47] Niklasson A (1991). An update of the Swedish reference standards for weight, length and head circumference at birth for given gestational age (1977–1981). Acta Paediatr. Scand..

[48] Karlberg, J., Luo, Z.C. & Albertsson-Wikland, K. Body mass index reference values (mean and SD) for Swedish children. *Acta Paediatr. (Oslo, Norway: 1992)* **90**, 1427–1434 (2001). 10.1111/j.1651-2227.2001.tb01609.x 11853342

[49] Høgberg, U. & Larsson, N. Early dating by ultrasound and perinatal outcome. A cohort study. *Acta Obstet. Gynecol. Scand.* **76**, 907–912 (1997). 10.3109/00016349709034900 9435727

[50] Wikland, K.A., Luo, Z.C., Niklasson, A. & Karlberg, J. Swedish population-based longitudinal reference values from birth to 18 years of age for height, weight and head circumference. *Acta Paediatr. (Oslo, Norway: 1992)* **91**, 739–754 (2002). 10.1080/08035250213216 12200898

[51] RCoreTeam. *R: A Language and Environment for Statistical Computing*. Vienna, Austria. https://www.R-project.org/ (2019).

[52] Bates, D., Bolker, B., & Walker, S. Fitting linear mixed-effects models using lme4. *J. Statist. Softw.* **67(1)**, 1–48 (2015).

[53] Hadley Wickham, L.H. *tidyr: Tidy Messy Data*. https://CRAN.R-project.org/package=tidyr (2019).

[54] Stef van Buuren, K.G.-O. mice: Multivariate imputation by chained equations in R. *J. Stat. Softw.* **45(3)**, 1–67 (2011).

[55] Wickham H (2016). ggplot2: Elegant Graphics for Data Analysis.

[56] Ludvigsson JF (2015). Ethical aspects of registry-based research in the Nordic countries. Clin. Epidemiol..

