# Supplementary material for: Associations between capillary glucose during pregnancy and childhood growth to the age of five: a cohort study
Source: Sci Rep. 2022 Feb 3;12:1832. doi: 10.1038/s41598-022-05821-8 (PMC8813989; doi:10.1038/s41598-022-05821-8)
Supplement: Supplementary file 1 — Supplementary Information. [file 41598_2022_5821_MOESM1_ESM.pdf]

Supplementary tables and figures

Associations between capillary Glucose during pregnancy and childhood growth to the age of five: A cohort study

Anna Österroos*, Linda Lindström, Per Wikman, Anna-Karin Wikström, Inger Sundström Poromaa, Fredrik Ahlsson

Department of Women's and Children's Health, Uppsala University, Sweden

* Corresponding author

Supplementary figure S1

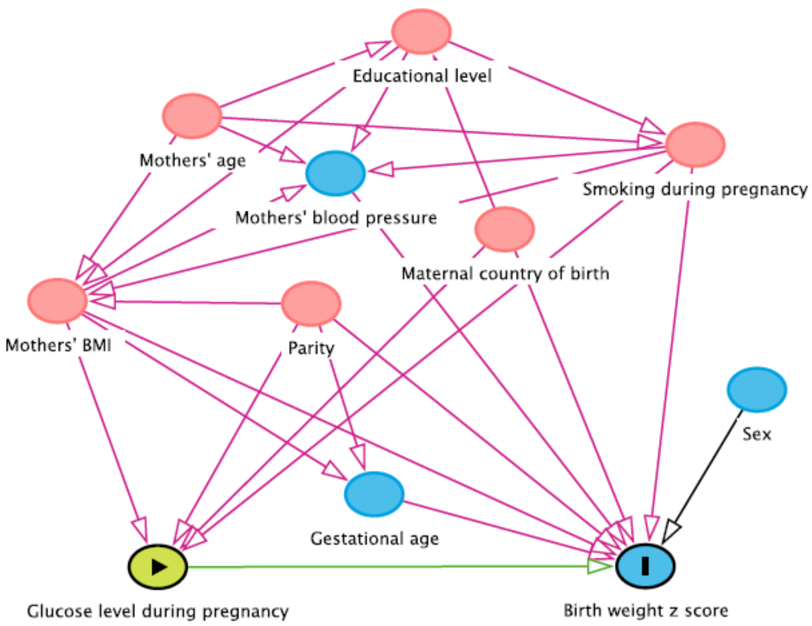

Drawing and Analyzing causal Diagram (DAG) for selection of covariates. Minimal sufficient adjustment sets for estimating the total effect of highest measured capillary glucose level during pregnancy on birth weight z score; Maternal country of birth, Mothers' BMI, Parity, Smoking during pregnancy. Version DAGitty v3.0.

Supplementary table S1. Weight z score trajectory from birth to five years of age.
Analysis was performed with linear mixed effect regression model with interactions between offspring age and maternal glucose quartiles to take repeated measurements in the same individuals in relation to highest maternal random capillary blood glucose level during pregnancy divided into quartiles into account. Glucose quartile 1 was reference for all interactions between maternal glucose quartiles and age of the children.

	**Unadjusted model** N = 27,145				**Adjusted model¤** N = 27,030			
	**β**	**95% CI** Lower	Upper	**p-value**	**β**	**95% CI** Lower	Upper	**p-value**
**Birth**								
Intercept	(-)0.01	-0.05	0.03	0.581	(-)0.84	-1.10	-0.59	<0.001*
Glucose quartile 1	ref	-	-	-	-	-	-	-
Glucose quartile 2	0.08	0.02	0.14	0.008*	0.07	0.01	0.13	0.014*
Glucose quartile 3	0.15	0.09	0.20	<0.001*	0.14	0.08	0.19	<0.001*
Glucose quartile 4	0.27	0.21	0.33	<0.001*	0.24	0.19	0.30	<0.001*
**1.5 years**								
Glucose quartile 1	0.04	0.00	0.08	0.080	0.04	0.00	0.08	0.071
Glucose quartile 2	(-)0.10	-0.15	-0.04	<0.001*	(-)0.10	-0.15	-0.04	0.001*
Glucose quartile 3	(-)0.13	-0.18	-0.07	<0.001*	(-)0.13	-0.19	-0.08	<0.001*
Glucose quartile 4	(-)0.30	-0.36	-0.25	<0.001*	(-)0.30	-0.36	-0.25	<0.001*
**3 years**								
Glucose quartile 1	(-)0.04	-0.09	0.00	0.063	(-)0.04	-0.09	0.00	0.068
Glucose quartile 2	(-)0.08	-0.14	-0.02	0.011*	(-)0.08	-0.14	-0.02	0.012*
Glucose quartile 3	(-)0.13	-0.19	-0.07	<0.001*	(-)0.13	-0.19	-0.07	<0.001*
Glucose quartile 4	(-)0.26	-0.32	-0.20	<0.001*	(-)0.26	-0.32	-0.20	<0.001*
**4 years**								
Glucose quartile 1	(-)0.05	-0.10	-0.01	0.030*	(-)0.05	-0.10	0.00	0.034*
Glucose quartile 2	(-)0.10	-0.16	-0.03	0.005*	(-)0.09	-0.16	-0.03	0.005*
Glucose quartile 3	(-)0.14	-0.20	-0.07	<0.001*	(-)0.14	-0.20	-0.08	<0.001*
Glucose quartile 4	(-)0.25	-0.31	-0.19	<0.001*	(-)0.25	-0.31	-0.19	<0.001*
**5 years**								
Glucose quartile 1	(-)0.07	-0.12	-0.02	0.010*	(-)0.07	-0.12	-0.01	0.011*
Glucose quartile 2	(-)0.11	-0.18	-0.04	0.003*	(-)0.11	-0.19	-0.04	0.003*
Glucose quartile 3	(-)0.13	-0.20	-0.06	<0.001*	(-)0.14	-0.21	-0.07	<0.001*
Glucose quartile 4	(-)0.25	-0.32	-0.18	<0.001*	(-)0.25	-0.32	-0.18	<0.001*

Sex and age independent standard deviation scores (z-scores) were calculated using the Swedish reference population.
N = Number of observations. β = β-value. 95% CI = 95% Confidence Interval.
* p <0.05
¤ Adjusted for parity, mothers' BMI at first maternal care visit, maternal country of birth and smoking in first and/or third trimester

Supplementary table S2. Height z score trajectory from birth to five years of age.
Analysis was performed with linear mixed effect regression model with interactions between offspring age and maternal glucose quartiles to take repeated measurements in the same individuals in relation to highest maternal random capillary blood glucose level during pregnancy divided into quartiles into account. Glucose quartile 1 was reference for all interactions between maternal glucose quartiles and age of the children.

	**Unadjusted model** N = 27,118				**Adjusted model ¤** N = 27,003			
	**β**	**95% CI** Lower	Upper	**p-value**	**β**	**95% CI** Lower	Upper	**p-value**
**Birth**								
Intercept	0.00	-0.04	0.04	0.965	(-)0.20	-0.47	0.07	0.140
Glucose quartile 1	ref	-	-	-	-	-	-	-
Glucose quartile 2	0.05	-0.01	0.11	0.135	0.04	-0.02	0.10	0.191
Glucose quartile 3	0.10	0.04	0.16	<0.001*	0.09	0.03	0.15	0.002*
Glucose quartile 4	0.15	0.09	0.21	<0.001*	0.14	0.08	0.20	<0.001*
**1.5 years**								
Glucose quartile 1	0.01	-0.03	0.05	0.629	0.01	-0.03	0.05	0.613
Glucose quartile 2	(-)0.07	-0.13	-0.01	0.019*	(-)0.07	-0.13	-0.01	0.020*
Glucose quartile 3	(-)0.13	-0.18	-0.07	<0.001*	(-)0.13	-0.18	-0.07	<0.001*
Glucose quartile 4	(-)0.18	-0.24	-0.12	<0.001*	(-)0.18	-0.24	-0.13	<0.001*
**3 years**								
Glucose quartile 1	(-)0.01	-0.06	0.03	0.560	(-)0.01	-0.06	0.03	0.571
Glucose quartile 2	(-)0.06	-0.12	0.01	0.082	(-)0.06	-0.12	0.01	0.090
Glucose quartile 3	(-)0.13	-0.19	-0.07	<0.001*	(-)0.13	-0.19	-0.07	<0.001*
Glucose quartile 4	(-)0.17	-0.23	-0.11	<0.001*	(-)0.17	-0.23	-0.11	<0.001*
**4 years**								
Glucose quartile 1	(-)0.02	-0.07	0.02	0.334	(-)0.02	-0.07	0.02	0.347
Glucose quartile 2	(-)0.03	-0.09	0.04	0.439	(-)0.03	-0.09	0.04	0.446
Glucose quartile 3	(-)0.11	-0.17	-0.05	<0.001*	(-)0.11	-0.17	-0.05	<0.001*
Glucose quartile 4	(-)0.14	-0.21	-0.08	<0.001*	(-)0.14	-0.21	-0.08	<0.001*
**5 years**								
Glucose quartile 1	(-)0.00	-0.05	0.05	0.974	(-)0.00	-0.05	0.05	0.935
Glucose quartile 2	(-)0.05	-0.12	0.03	0.219	(-)0.04	-0.12	0.03	0.250
Glucose quartile 3	(-)0.14	-0.21	-0.07	<0.001*	(-)0.14	-0.21	-0.07	<0.001*
Glucose quartile 4	(-)0.16	-0.23	-0.09	<0.001*	(-)0.16	-0.23	-0.09	<0.001*

Sex and age independent standard deviation scores (z-scores) were calculated using the Swedish reference population
N = Number of observations. β = β-value. 95% CI = 95% Confidence Interval.
* p <0.05
¤ Adjusted for parity, mothers' BMI at first maternal care visit, maternal country of birth and smoking in first and/or third trimester

Supplementary table S3. BMI z score trajectory from birth to five years of age.
Analysis was performed with linear mixed effect regression model with interactions between offspring age and maternal glucose quartiles to take repeated measurements in the same individuals in relation to highest maternal random capillary blood glucose level during pregnancy divided into quartiles into account. Glucose quartile 1 was reference for all interactions between maternal glucose quartiles and age of the children.

	**Unadjusted model** N = 27,098				**Adjusted model ¤** N = 26,983			
	**β**	**95% CI**		**p-value**	**β**	**95% CI**		**p-value**
		Lower	Upper			Lower	Upper	
**Birth**								
Intercept	0.01	-0.03	0.05	0.682	(-)1.04	-1.28	-0.79	<0.001*
Glucose quartile 1	ref	-	-	-	-	-	-	-
Glucose quartile 2	0.07	0.01	0.13	0.016*	0.07	0.01	0.13	0.023*
Glucose quartile 3	0.13	0.07	0.19	<0.001*	0.12	0.06	0.18	<0.001*
Glucose quartile 4	0.26	0.21	0.32	<0.001*	0.24	0.18	0.29	<0.001*
**1.5 years**								
Glucose quartile 1	0.02	-0.03	0.06	0.505	0.02	-0.03	0.06	0.457
Glucose quartile 2	(-)0.08	-0.14	-0.01	0.021*	(-)0.08	-0.14	-0.01	0.021*
Glucose quartile 3	(-)0.08	-0.14	-0.02	0.014*	(-)0.08	-0.15	-0.02	0.010*
Glucose quartile 4	(-)0.27	-0.34	-0.21	<0.001*	(-)0.28	-0.34	-0.21	<0.001*
**3 years**								
Glucose quartile 1	(-)0.07	-0.12	-0.02	0.004	(-)0.07	-0.12	-0.02	0.005*
Glucose quartile 2	(-)0.07	-0.14	0.00	0.060	(-)0.07	-0.14	0.00	0.060
Glucose quartile 3	(-)0.07	-0.14	0.00	0.039*	(-)0.08	-0.14	-0.01	0.031*
Glucose quartile 4	(-)0.23	-0.30	-0.16	<0.001*	(-)0.23	-0.30	-0.16	<0.001*
**4 years**								
Glucose quartile 1	(-)0.08	-0.13	-0.02	0.004*	(-)0.08	-0.13	-0.02	0.005*
Glucose quartile 2	(-)0.12	-0.19	-0.04	0.002*	(-)0.12	-0.19	-0.04	0.002*
Glucose quartile 3	(-)0.11	-0.18	-0.04	0.002*	(-)0.12	-0.19	-0.05	0.001*
Glucose quartile 4	(-)0.25	-0.32	-0.18	<0.001*	(-)0.25	-0.32	-0.18	<0.001*
**5 years**								
Glucose quartile 1	(-)0.12	-0.18	-0.06	<0.001*	(-)0.12	-0.18	-0.06	<0.001*
Glucose quartile 2	(-)0.12	-0.20	-0.03	0.006*	(-)0.12	-0.20	-0.04	0.005*
Glucose quartile 3	(-)0.08	-0.16	0.00	0.048*	(-)0.09	-0.17	-0.01	0.033*
Glucose quartile 4	(-)0.23	-0.31	-0.15	<0.001*	(-)0.23	-0.31	-0.15	<0.001*

Sex and age independent standard deviation scores (z-scores) were calculated using the Swedish reference population.
N = Number of observations. β = β-value. 95% CI = 95% Confidence Interval.
* p <0.05
¤ Adjusted for parity, mothers' BMI at first maternal care visit, maternal country of birth and smoking in first and/or third trimester

Supplementary table S4 Comparison between first and highest capillary glucose level. Number of observations and percentage of total population where the highest measured random capillary blood glucose level were noted at the first maternal health care visit before pregnancy week 13.

First glucose mean (mmol/L)	5.1
Highest glucose mean (mmol/L)	6.2
First = Highest glucose (N (%))	
Yes	747 (9.4)
No	3071 (38.7)
Missing data for first glucose level	4127 (51.2)
Yes (without missing data)	747 (19.8)
No (without missing data)	3027 (80.2)
